# Understanding the Binding Transition State After the Conformational Selection Step: The Second Half of the Molecular Recognition Process Between NS1 of the 1918 Influenza Virus and Host p85β

**DOI:** 10.3389/fmolb.2021.716477

**Published:** 2021-07-08

**Authors:** Alyssa Dubrow, Iktae Kim, Elias Topo, Jae-Hyun Cho

**Affiliations:** Department of Biochemistry and Biophysics, Texas A&M University, College Station, TX, United States

**Keywords:** nonstructural protein 1, molecular recognition, transition state, protein-protein interaction, conformational selection, influenza virus

## Abstract

Biomolecular recognition often involves conformational changes as a prerequisite for binding (i.e., conformational selection) or concurrently with binding (i.e., induced-fit). Recent advances in structural and kinetic approaches have enabled the detailed characterization of protein motions at atomic resolution. However, to fully understand the role of the conformational dynamics in molecular recognition, studies on the binding transition state are needed. Here, we investigate the binding transition state between nonstructural protein 1 (NS1) of the pandemic 1918 influenza A virus and the human p85β subunit of PI3K. 1918 NS1 binds to p85β *via* conformational selection. We present the free-energy mapping of the transition and bound states of the 1918 NS1:p85β interaction using linear free energy relationship and ϕ-value analyses. We find that the binding transition state of 1918 NS1 and p85β is structurally similar to the bound state with well-defined binding orientation and hydrophobic interactions. Our finding provides a detailed view of how protein motion contributes to the development of intermolecular interactions along the binding reaction coordinate.

## Introduction

Understanding the role of conformational dynamics remains a central topic in the mechanistic study of biomolecular recognition (Boehr et al., 2009; Wand and Sharp, 2018). A particular interest is how the conformational change is coupled to the binding process. For example, a conformational adaptation of proteins may occur via conformational selection or induced-fit (Changeux and Edelstein, 2011; Vogt and Di Cera, 2012). To distinguish between the two limiting models, diverse kinetic approaches have been proposed (Hammes et al., 2009; Vogt and Di Cera, 2012; Gianni et al., 2014; Paul and Weikl, 2016). Moreover, recent advancement of structural biology techniques, especially NMR relaxation dynamics, has enabled detailed characterization of the intrinsic conformational dynamics in the pre-binding step (Sugase et al., 2007; Lange et al., 2008; Cho et al., 2010; Kovermann et al., 2017; Sekhar et al., 2018; Cho et al., 2020). Thus, combining the kinetic and structural dynamics approaches has deepened our understanding of the functional role of intrinsic conformational dynamics in molecular recognition (Phillips et al., 2013; Chakrabarti et al., 2016; Cho et al., 2020).

Despite much progress, however, there remain outstanding questions as to the role of conformational dynamics in binding processes. For example, many protein-protein interaction (PPI) interfaces consist of heterogeneous regions in which some parts require a major conformational change while other parts only need a minor or no conformational change. Then, which region contributes to the binding transition state more significantly? In addition, what is the role of the “conformationally selected” residues in the binding step? Do they significantly contribute to stabilizing the binding transition state (TS), or is the conformational change needed only to avoid a steric clash during binding? To address these questions, we focus, in the present study, on the molecular recognition of NS1 from the 1918 influenza A virus (IAV).

**FIGURE 1:**
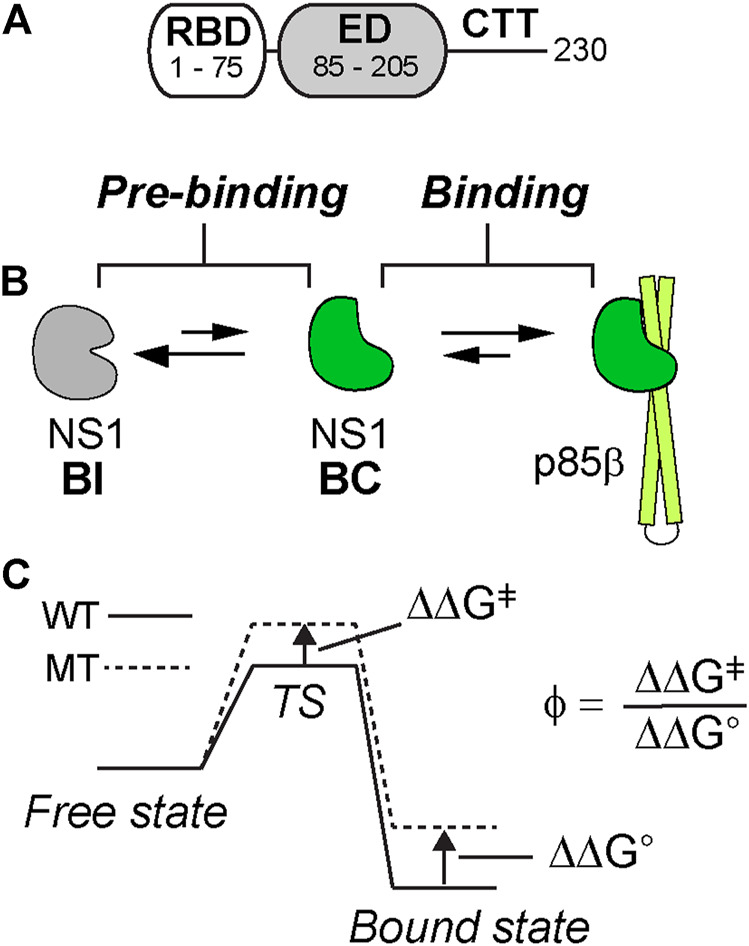
**(A)** Domain organization of 1918 NS1 **(B)** Binding scheme between 1918 NS1 and p85β. BI and BC represent binding-incompetent and –competent states, respectively **(C)** Diagram showing graphical definitions of ΔΔG°, ΔΔG^ǂ^, and ϕ value.

The 1918 IAV caused the worst flu pandemic (a. k. a. Spanish flu) in recorded human history (Taubenberger, 2006; Taubenberger and Morens, 2006). NS1 of IAV is a major virulence factor and is responsible for suppressing host innate immune responses during the infection cycle (Min and Krug, 2006; Das et al., 2008; Hale et al., 2010a; Krug, 2015). Moreover, it was indicated that 1918 NS1 is an effective interferon antagonist (Basler et al., 2001; Geiss et al., 2002). Structurally, IAV NS1 consists of an RNA-binding domain (RBD), an effector domain (ED), followed by a structurally disordered C-terminal tail (Figure 1A) (Carrillo et al., 2014; Hale, 2014). NS1-ED is a particularly interesting domain because it binds to many host factors involved in innate immune responses, such as phosphoinositide 3-kinase (PI3K) (Hale et al., 2010b), 30-kDa cleavage and polyadenylation specificity factor 30 (CPSF30) (Das et al., 2008), and protein kinase R (PKR) (Bergmann et al., 2000).

Binding of 1918 NS1 to PI3K results in the inhibition of apoptosis or triggering cation conductance in the infected lung epithelium (Ehrhardt et al., 2007; Gallacher et al., 2009). Interestingly, we have recently found that the free 1918 NS1 has a p85β binding-incompetent (BI) conformation as a major population and undergoes a conformational change to form a transiently populated, binding-competent (BC) form in sub-millisecond timescale (Figure 1B) (Cho et al., 2020). Therefore, the binding mechanism is best characterized as a conformational selection model. Moreover, NS1 exploits most of its surface area in order to bind multiple host proteins, suggesting that the conformational change in the p85β-binding site allosterically affects other bindings (Cho et al., 2020).

Here we characterize the binding TS between 1918 NS1-ED and p85β using linear free energy relationship (LFER) (Leffler, 1953) and ϕ-value analyses (Figure 1C) (Goldenberg et al., 1989; Fersht and Sato, 2004) combined with Ala-scanning. We find that the binding TS is structurally similar to the bound state. We identify that a hydrophobic cluster surrounding a buried hydrogen bond is critical for stabilizing both the transition and bound states. Our result also suggests that the binding process involves characteristics of both conformational selection and induced fit models.

## Results

### Selection Strategy of Interface Residues for Mutagenesis

**FIGURE 2:**
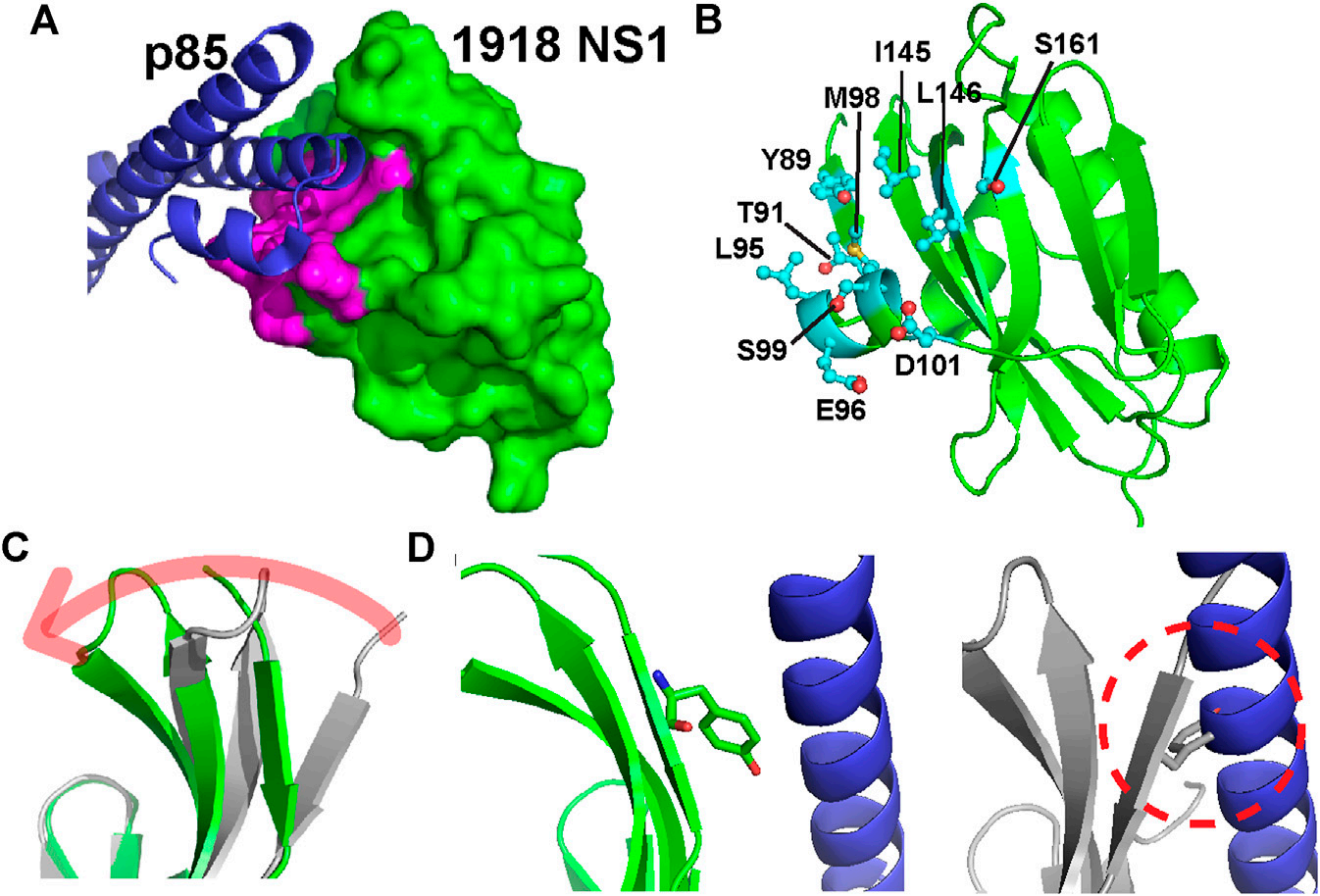
**(A)** Structure of the 1918 NS1:p85β complex (PDB ID: 6U28). Binding interface residues on 1918 NS1 included in the present study are shown in magenta **(B)** The Ala-substituted residues on 1918 NS1 are shown in a stick model. The orientation of 1918 NS1 is the same as in panel A **(C)** Overlay of 1918 NS1 structures of the free (gray) and p85β-bound (green) states. Arrow shows the direction of conformational change upon binding to p85β **(D)** Conformations of Y89 in the bound **(left panel)** and free **(right panel)** states of 1918 NS1. Dashed circle shows the hypothetical steric clash of Y89 with p85β when free 1918 NS1 is docked to the complex structure.

The 1918 NS1-ED (hereinafter NS1) residues on the p85β-binding interface are identified by the change in solvent accessible surface area of individual residues upon complexation (ΔSASA_bind_) using the structure of the complex (PDB ID: 6U28) (Figure 2A). The total interface area was 1,658.15 Å^2^; changes in the polar and apolar surface area were 531.35 Å^2^ and 1,126.81 Å^2^, respectively. Thus, the binding interface is mainly hydrophobic. The interface includes 20 residues on 1918 NS1, among which we selected ten residues for Ala-scanning mutagenesis (Figure 2B). The selected residues involve all hydrophobic residues and polar and charged residues whose ΔSASA_bind_ is larger than 20 Å^2^ between the free and complex structures. The selected residues exhibit varying degrees of conformational changes upon binding to p85β (Figure 2C). The most significant change occurs in the residues of β1 strand. For example, χ1 angle of Y89 in the β1 strand changes from −54.8° in the free state to 177.2° in the bound state (Figure 2D). It is also important to note that Ala-substitution of the selected residues in the present study was previously applied to NS1 of Puerto Rico 8 (PR8) IAV strain for a cell-based study on its interaction with p85β (Lopes et al., 2017). Thus, the present study on 1918 NS1 enables the comparison with the result of cell-based assays (see Discussion).

### Energetic Contribution of Interface Residues to the Binding Affinity

**FIGURE 3:**
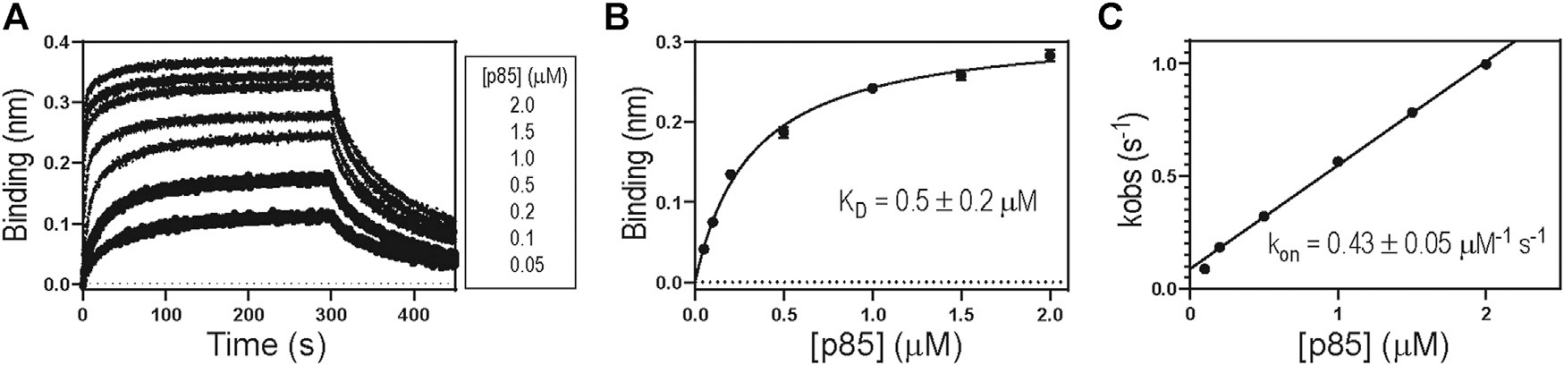
**(A)** A representative BLI sensorgram showing binding between 1918 NS1 WT and p85β. Representative **(B)** binding isotherm and **(C)** binding kinetics between 1918 NS1 WT and p85β.

In the present study, we employed 1918 NS1 (residues 80-205) in which the C-terminal tail (CTT) is deleted (Figure 1A). The crystal structure of the 1918 NS1:p85β complex showed that the CTT does not interact with p85β (Cho et al., 2020). Indeed, we found that the K_D_ value of NS1 without CTT is highly similar to the previously reported value of NS1 with CTT (Figures 3A,B) (Cho et al., 2020). In addition, we incorporated W187R substitution to prevent homodimerization of 1918 NS1 (Aramini et al., 2011); thus, wild type (WT) in the present study corresponds to 1918 NS1 W187R. All mutants were prepared on W187R background. We have previously shown that W187 is exposed to solvent, located on the opposite side of the p85β-binding site, and W187R mutation does not affect the binding to p85β (Cho et al., 2020).

**FIGURE 4:**
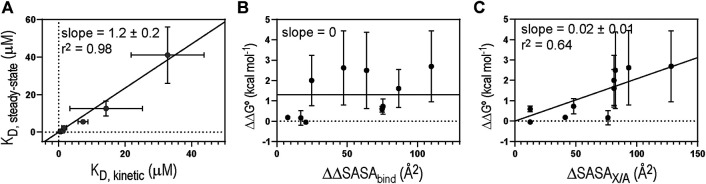
**(A)** Correlation between K_D_ values derived by steady-state and kinetic methods. Solid diagonal line represents the best linear regression curve. Plots of **(B)** ΔΔG° vs. ΔΔSASA_bind_ and **(C)** ΔΔG° vs ΔSASA_X/A_. Solid lines represent the linear regression curves selected by hypothesis testing between the null (slope = 0) and alternative (slope ≠ 0) hypotheses. Critical α value was 0.05 for the hypothesis test (F-test).

Table 1 shows the biolayer interferometry (BLI)-measured binding parameters of 1918 NS1 WT and mutants involved in this study. We noticed that some mutants bind to p85β with a dramatically low affinity, and consequently, K_D_ values measured by the BLI steady-state method was not reliable. In contrast, mutants without the large change in binding affinity showed a good agreement between kinetics-derived and steady-state-derived K_D_ values. (Figure 4A). For the consistency, we used K_D_ values measured by the kinetic method (i.e., K_D_ = k_off_/k_on_). A linear relationship between k_obs_ and (p85β) suggests that an apparent two-state binding scheme is applicable to calculate k_on_ and k_off_ values (Figure 3C). We also confirmed that the BLI-derived K_D_ is comparable with the true equilibrium value obtained by ITC (Supplementary Figure 1).

**TABLE 1:** Binding parameters of 1918 NS1 wild type and mutants.

Proteins	ϕ-values^a^	K_D_ (μM)^a^ ^,^ ^b^	k_on_ (μM^−1^ s^−1^)^b^	k_off_ (s^−1^)^b^
Wild type	–	0.5 ± 0.1	0.43 ± 0.05	0.23 ± 0.02
Y89A	0.7 ± 0.2	46 ± 21	0.02 ± 0.01	1.0 ± 0.3
T91A	N. D^c^	0.7 ± 0.3	0.4 ± 0.2	0.3 ± 0.1
L95A	0.6 ± 0.1	7 ± 2	0.09 ± 0.01	0.7 ± 0.1
E96A	N. D^c^	0.6 ± 0.1	0.24 ± 0.02	0.15 ± 0.03
M98A	0.7 ± 0.1	41 ± 13	0.02 ± 0.01	0.8 ± 0.1
S99A	0.2 ± 0.2	1.3 ± 0.3	0.34 ± 0.04	0.5 ± 0.1
D101A	0.5 ± 0.5	1.7 ± 0.7	0.2 ± 0.1	0.4 ± 0.1
I145A	0.8 ± 0.1	33 ± 11	0.02 ± 0.01	0.6 ± 0.1
L146A	0.6 ± 0.4	14 ± 11	0.05 ± 0.04	0.8 ± 0.2
S161A	N. D^c^	0.4 ± 0.2	0.5 ± 0.1	0.2 ± 0.1

a Uncertainties represent propagated errors.

b All values are represented by average ± standard deviation of 3 repeats. Average values were rounded to have the same number of significant figures with standard deviation.

c Not Determined due to small ΔΔG°.

Overall, the Ala-substitution of hydrophobic interface residues significantly affected the binding affinity. However, we found no statistically significant correlation between the change in ΔSASA_bind_ upon Ala-substitution of individual residues (ΔΔSASA_bind_) and the change in K_D_ (ΔΔG°) (Figure 4B). Rather, we found that ΔΔG° correlates with the change in SASA of free 1918 NS1 by Ala-substitution of individual residues (ΔSASA_X/A_, where X and A correspond to any residues and Ala, respectively) (Figure 4C). These results indicate that hydrophobic interactions and the extent of packing at the interface might play a critical role in the binding affinity of the 1918NS1:p85β complex.

### Characterization of the Binding Transition State

**FIGURE 5:**
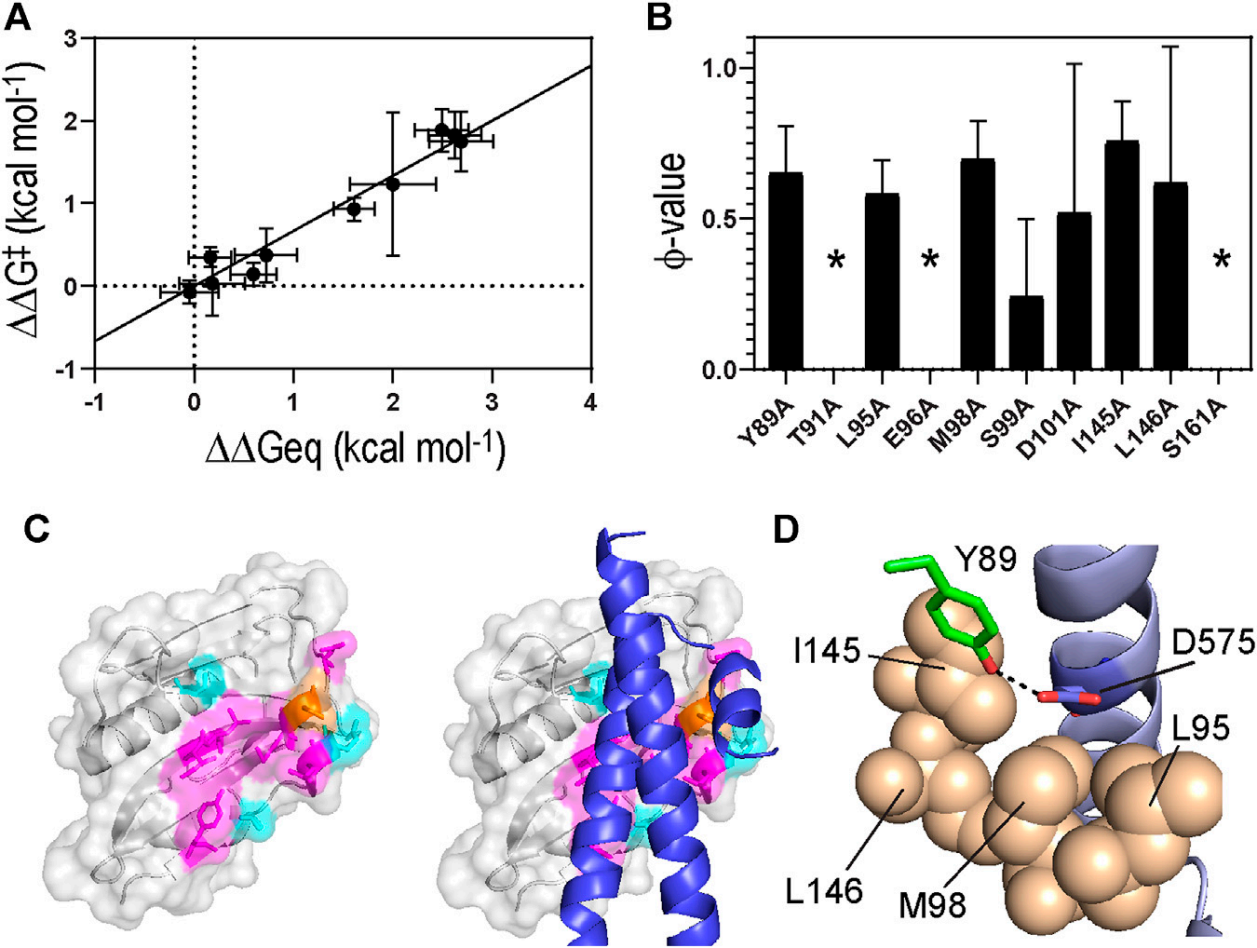
**(A)** A linear free energy plot showing ΔΔG° against ΔΔG^ǂ^ **(B)** ϕ-values obtained for 1918 NS1 binding to p85β. Asterisks correspond to the residues whose ϕ-values were not determined due to small ΔΔG° **(C)** **(Left)** Color-coded ϕ-values on the surface of 1918 NS1. Residues shown in magenta and orange have high-intermediate (0.5–0.8) and low (<0.5) ϕ-values, respectively. Residues showed small ΔΔG° (<0.5 kcal mol^−1^) upon Ala substitution are shown in cyan **(Right)** Structure of p85β is superimposed on the 1918 NS1 structure **(D)** The hydrogen bond between Y89 of NS1 and D575 of p85β. Hydrophobic residues surrounding the hydrogen bond are shown in sphere representation.

We first assessed the development of global intermolecular interactions at the TS using LFER analysis (Figure 5A). The slope of the LFER plot is often called Leffler α-value and is an estimate of the TS position along a reaction coordinate, although α-value can differ from the true TS position in a complicated reaction (Leffler, 1953; Fersht, 2004). All data points in the LFER plot exhibited good linearity, suggesting that the binding reaction occurs through a major TS (Fersht, 2004). In contrast, when binding occurs through highly distinct, multiple TSs, the LFER plot can show a non-linear relationship (Sánchez and Kiefhaber, 2003; Bokhovchuk et al., 2020).

The Leffler α-value of the 1918 NS1:p85β interaction was measured to be 0.67 ± 0.06 (Figure 5A), indicating that the binding TS is located at a relatively later stage in the reaction coordinate. This result also suggests that the overall TS structure is similar to that of the bound state. 1918 NS1 forms the BC (i.e., bound-like) conformation that is, a marginally populated, on-pathway intermediate in the binding reaction coordinate (Cho et al., 2020). Therefore, it is reasonable that the binding TS has a similar structure to the bound state.

To characterize the binding TS on a residue level, we conducted a ϕ-value analysis (Matouschek et al., 1989). While LFER reveals the position of the TS along with the reaction coordinate, ϕ-value analysis provides the extent of native (or bound)-like interaction of individual side chains at the TS relative to the native state (Fersht, 2004). Although the ϕ-value analysis was originally developed for studying the protein folding process, it can also characterize the TS of PPIs (Horn et al., 2009; Schreiber et al., 2009).

A ϕ-value close to 0 suggests the mutated residue does not form intermolecular interaction at the TS; i.e., the interaction at the TS is similar to that in the free state. On the contrary, a ϕ-value close to 1 suggests that the mutated residue develops a TS interaction similar to that in the bound state. Intermediate ϕ-values are often interpreted as partial bound-like interaction, although alternative interpretations are possible (Fersht and Sato, 2004; Cho and Raleigh, 2006; Cho et al., 2014). In this analysis, we only included mutations of which ΔΔG° (= ΔG°_WT_–ΔG°_MT_) is larger than 0.5 kcal mol^−1^ to avoid artifactual ϕ-values (Fersht and Sato, 2004); seven out of the ten mutants included in this study showed a change in binding free energy of >0.5 kcal mol^−1^ (Table 1 and Figure 5B).

The Ala-substitution of hydrophobic residues showed significant effects on both binding affinity and kinetics, indicating their primary role in the binding process. The average ϕ-value of the hydrophobic residues was 0.66 (Table 1 and Figure 5B). Structurally, the hydrophobic residues are clustered on the binding surface of 1918 NS1 (Figure 5C). The cluster of the hydrophobic residues with high-intermediate ϕ-values (0.5–0.7) suggests cooperativity of the residues in the binding TS. Moreover, the hydrophobic clustering seems critical for the functionally critical interaction mediated by Y89 (vide infra).

Y89 is of special interest because the residue is highly conserved in NS1 proteins of almost all human IAVs (Hale et al., 2006; Cho et al., 2020). The importance of this evolutionarily conserved residue was well-demonstrated by functional studies (Hale et al., 2006; Hale et al., 2010b). The side chain of Y89 undergoes a noticeable conformational change upon binding to p85β (Figure 2C), without which 1918 NS1 would sterically clash with p85β (Figure 2D). Y89A resulted in large ΔΔG° and ΔΔG^ǂ^, yielding a ϕ-value of 0.65; this indicates that Y89 forms a substantial interaction at the binding TS. In the complex, Y89 forms a buried hydrogen bond to D575 of p85β in the middle of the hydrophobic cluster at the binding interface (Figure 5D). We previously showed that deleting the hydroxyl group by Y89F also impaired the binding significantly (Cho et al., 2020).

The large energetic contribution of the hydrogen bond to the binding energetics might be due to the burial of the bond in the hydrophobic interface. The strength of a hydrogen bond can be significantly higher in the hydrophobic environment than on the solvent-exposed surface (Hendsch and Tidor, 1994; Albeck et al., 2000; Dai et al., 2019). Substantial ΔΔG^ǂ^ of Y89A indicates that hydrophobic residues already surround the hydrogen bond at the binding TS similarly to the bound state. Moreover, while hydrophobic interactions are generally non-specific, the buried hydrogen bond might play a role in binding orientation. In other words, the TS structure might not be randomly oriented because of the Y89^NS1^-D575^p85^ interaction.

In contrast to hydrophobic residues, Ala-mutagenesis of polar and charged residues exhibited a marginal effect on both binding affinity and kinetics. Only two residues, S99A and D101A, changed ΔΔG° >0.5 kcal mol^−1^. Consequently, ϕ-values of other polar and charged residues were not calculated due to small ΔΔG°. However, this result indicates that the energetic contribution of polar and charged residues to the binding affinity and kinetics is generally small.

However, D101 was exceptional, whose ϕ-value was measured to be 0.5. Although its uncertainty seems large, additional statistical test supports that the following structural interpretation of the ϕ-value is plausible (Supplementary Text). The side chain of D101 interacts with the backbone amide of Q588 of p85β in the bound state (Supplementary Figure 2). Considering the reasonably high ϕ-values of Y89 and D101, the specific binding orientation seems to be well-defined at the TS (Supplementary Figure 2). Interestingly, we previously showed that the 1918NS1:p85β interaction is not significantly affected by a high salt concentration (Dubrow et al., 2020). Although this seems contrasting with the result of D101A, the difference is reconciled by considering that the ϕ-value is a relative parameter. Namely, the absolute free energy change upon D101A mutation is significantly less than those by mutations of hydrophobic residues. Moreover, a salt-dependent study is not effective in screening short-range electrostatic interactions (Luisi et al., 2003).

## Discussion

Lopes et al. reported the result of Ala-scanning of p85β-binding interface on PR8 NS1 using cell-based assays (Lopes et al., 2017). Because the p85β-binding interface is completely conserved between PR8 and 1918 NS1s, it is worth comparing their result with ours. The authors found that Y89A, M98A, and I145A decreased binding affinity to p85β by 10 to 100-fold compared to that of PR8 NS1-WT. Additionally, L95A, S99A, D101A, and L146A were identified to decrease the affinity by 2 to 3-fold relative to wild type. These results are remarkably consistent with our observation. Therefore, our result is likely to provide mechanistic details of the functional molecular recognition by 1918 NS1.

Our result indicates that hydrophobic interaction is a major driving force for stabilizing both TS and bound state between 1918 NS1 and p85β. Similar results were reported for other PPIs. For example, Bokhovchuk et al. showed that the binding TS between YAP (Yes-associated protein) and TEAD (TEA/ATTS domain) forms a cluster of hydrophobic residues with high ϕ-values (Bokhovchuk et al., 2020). Horn et al. showed that the binding TS between hGHs (human growth hormones) and their receptor (hGHR) contains a cluster of hydrophobic residues exhibiting high ϕ-values (Horn et al., 2009). The authors also found that the TS structure adopts a native-like orientational specificity. This is analogous to ours; moderately high ϕ-values of Y89^NS1^ and D101^NS1^ indicate that the binding TS has a specific binding orientation similar to that of the bound state.

We also note the cases contrasting to our result. Extensive studies on barnase-barstar and TEM1-BLIP interactions demonstrated that hydrophobic residues have low ϕ-values (<0.1), while charged residues located outside the binding site have high ϕ-values (>0.8). Interestingly, charged residues within the binding site tend to have mixed values (0–0.6) (Schreiber et al., 2009). Remarkably, some of the experimental results were reproduced in recent MD simulations. Pan et al. conducted MD simulations of five PPIs, including barnase-barstar, TEM1-BLIP, and Ras-RAF (Pan et al., 2019). Their simulation result showed that the TSs of the PPIs interfaces are highly hydrated with a low level of native contacts (<20%), implying weak hydrophobic interactions at the TS.

Our previous study revealed a dynamic equilibrium of 1918 NS1 between BI and BC conformers in the pre-binding step (Cho et al., 2020). Thus, 1918 NS1 binds to p85β most likely via conformational selection. However, it is also reasonable to assume that the BC conformer might need further conformational adjustment along the binding process. Our present result demonstrates that p85β-binding interface on 1918 NS1 form a significant level of bound-like interactions at the TS. Nevertheless, it is worth noting that most of the polar and charged residues in the interface only have weak intermolecular interactions at the TS, suggesting the need for further conformational fine-tuning after the TS. In this respect, the 1918 NS1:p85β interaction might be viewed as a combination of conformational selection and induced fit. On a similar note, Wlodarski, and Zagrovic reported that ubiquitin undergoes conformational selection followed by induced fit in the binding process (Wlodarski and Zagrovic, 2009).

Interestingly, studies on the PPIs mediated by IDPs also demonstrated the coupling of conformational selection and induced fit mechanisms. For example, ϕ-value and LFER analyses revealed that the binding TS of c-Myb:KIX complex has a high content of bound-like structure (Giri et al., 2013). A detailed NMR study further showed that the Nand C-terminal region of c-Myb binds to KIX through conformational selection and induced fit mechanisms (Arai et al., 2015), respectively. Similar coupling of the two binding mechanisms was also observed for the binding of the YAP:TEAD complex (Bokhovchuk et al., 2020).

In conclusion, the present study provides insights into how conformational selection plays the dual role along a binding reaction, avoiding steric clash by populating binding-competent form and stabilizing the binding TS. Testing the generality of our observation in other conformational selection-based PPIs would be highly desirable in the future. In addition, we observed that the binding of 1918 NS1 and p85β is best characterized by a coupled mechanism of conformational selection and induced fit, which is found in the binding of IDPs. Although the present study does not include a direct measurement of protein motion, the induced fit model implies protein motion or conformational adaptation after the binding transition state. Our previous study showed that 1918 NS1 undergoes conformational dynamics between BI and BC states in the pre-binding step (Cho et al., 2020). Taken together, we infer that protein motions play a key role in both pre-binding and binding steps of 1918 NS1 and p85β.

## Materials and Methods

### Protein Sample Preparation

1918 NS1 (residues 80-205) WT and mutants involved in this study were expressed in BL21 (DE3) *Escherichia coli* cells with His_6_ and SUMO tags and purified by Ni^2+^ NTA column followed by gel-filtration chromatography. The p85β (residues 435-599) was also expressed in BL21 (DE3) *E. coli* and purified in the same way as 1918 NS1 proteins.

### Isothermal Titration Calorimetry

Data was acquired at 25°C using a MicroCal PEAQ-ITC (Malvern Panalytical) instrument. 100 μM of p85β was in the syringe and 10 μM NS1 was in the cell. Both samples were prepared in the same buffer; 20 mM sodium phosphate (pH 7.0), 1 mM TCEP, and 100 mM NaCl. The K_D_ value was directly obtained from fitting the data to a 1:1 binding model.

### Binding Parameters and ϕ-Values

The binding affinity between 1918 NS1 and p85β was measured at 25°C using an Octet RED biolayer interferometer (Pall ForteBio). His_6_-SUMO-tagged NS1 proteins were immobilized on the Ni-NTA biosensor. The buffer contains 20 mM sodium phosphate (pH 7.0). 150 mM NaCl, 1% bovine serum albumin, and 50 mM imidazole. Representative sensorgrams of binding between all 1918 NS1 mutants and p85β are shown in Supplementary Figure 3. All measurements were repeated at least three times. Reported values are the average and standard error of the mean calculated using the repeated measurements.

In BLI binding experiments, the 1918 NS1:p85β interaction exhibited bi-phasic association and dissociation curves due to non-specific binding. The k_obs_ was measured by fitting a fast-phase of the association curve using a single-exponential function. The k_on_ was calculated using linear regression of p85-dependent association rate [k_obs_ vs. (p85β)]. The k_off_ was calculated from the direct measurement of the fast-phase in a dissociation curve.

ϕ-values were calculated by dividing ΔΔG^ǂ^ by ΔΔG° which were calculated using following equations:

$$\Delta \Delta G^{{}^{\circ}}=\Delta G_{Kd}^{WT}-\Delta G_{Kd}^{MT}=-RT\ln\left(K_{D}^{WT}/K_{D}^{MT}\right)$$ (1)

$$\Delta \Delta G^{‡}=\Delta G_{kon}^{MT}-\Delta G_{kon}^{WT}=-RT\ln\left(k_{on}^{MT}/k_{on}^{WT}\right)$$ (2)

## Data Availability

The datasets analyzed in this study can be found in online repositories. The names of the repository/repositories and accession number(s) can be found in the Supplementary Material and http://www.wwpdb.org/, 6U28.
