# Supplementary material for: Understanding the Binding Transition State After the Conformational Selection Step: The Second Half of the Molecular Recognition Process Between NS1 of the 1918 Influenza Virus and Host p85β
Source: Front Mol Biosci. 2021 Jul 8;8:716477. doi: 10.3389/fmolb.2021.716477 (PMC8296144; doi:10.3389/fmolb.2021.716477)
Supplement: Supplementary file 1 [file DataSheet1.pdf]

Supplementary Material

Dubrow et al.

Understanding the binding transition state after the conformational selection step: the second half of the molecular recognition process between NS1 of the 1918 influenza virus and host p85β

Supplementary Text

To test the validity of the ϕ-value of D101A, we calculated the ϕ-value using two additional methods as follows. First, it should be noted that phi-value can be calculated by either k_{on} or k_{off}, as long as the binding kinetics can be presented in a two-state process. Moreover, k_{on} and k_{off} values were estimated independently in our study.

So, we calculated the dissociation ϕ-value (ϕ_{off}) using k_{off} values. Note that, in the manuscript, we reported the ϕ value for the association process (ϕ_{on}), which is equivalent to 1- ϕ_{off} (i.e., $\phi_{on} = 1 - \phi_{off}$). Our calculation yielded $\phi_{off} = 0.3 \pm 0.5$; thus, $\phi_{on} = 0.7 \pm 0.5$. Second, we calculated ϕ (i.e., ϕ_{on}) using steady-state K_d values measured by BLI. As mentioned in the manuscript, K_d values of some mutants were measured by both steady-state and kinetic methods (Figure 4A), and D101A was one of them. $\Delta\Delta G°$ estimated by steady-state method was 0.94 ± 0.36 kcal mol^{-1}, yielding $\phi = 0.4 \pm 0.4$. Taken together, the average (and standard error of the mean) of the three estimated ϕ values is 0.5 (± 0.1). The standard error of the mean is calculated standard deviation of the three ϕ-values (= 0.21) divided by the square root of the number of measurements (= $3^{0.5}$).

This indicates that the ϕ-value of D101A can be interpreted with a reasonable statistical significance. Moreover, the new average phi value is consistent with our inference described in the manuscript; i.e., D101 forms about 50% of bound state interaction in the binding transition state, thus, contributes to specific binding orientation together with Y89.

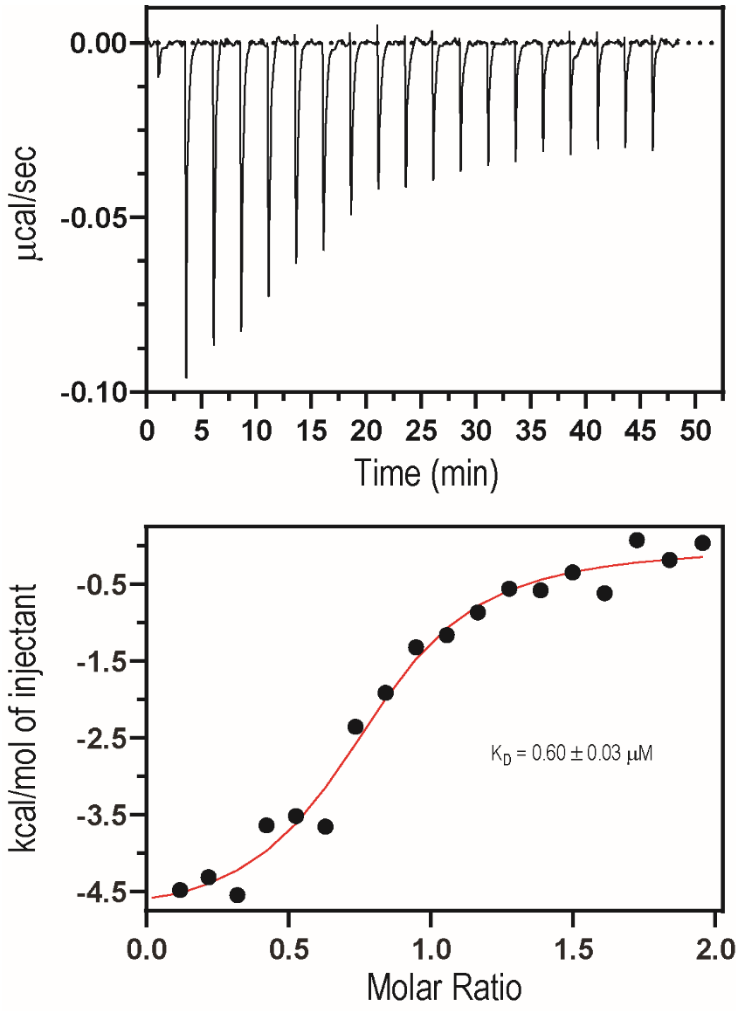

Supplementary Figure 1. Isothermal titration calorimetry (ITC) traces and binding isotherm of the titration of 1918 NS1 into p85β. Solid line represents the best fit to the binding isotherm with a 1:1 binding model.

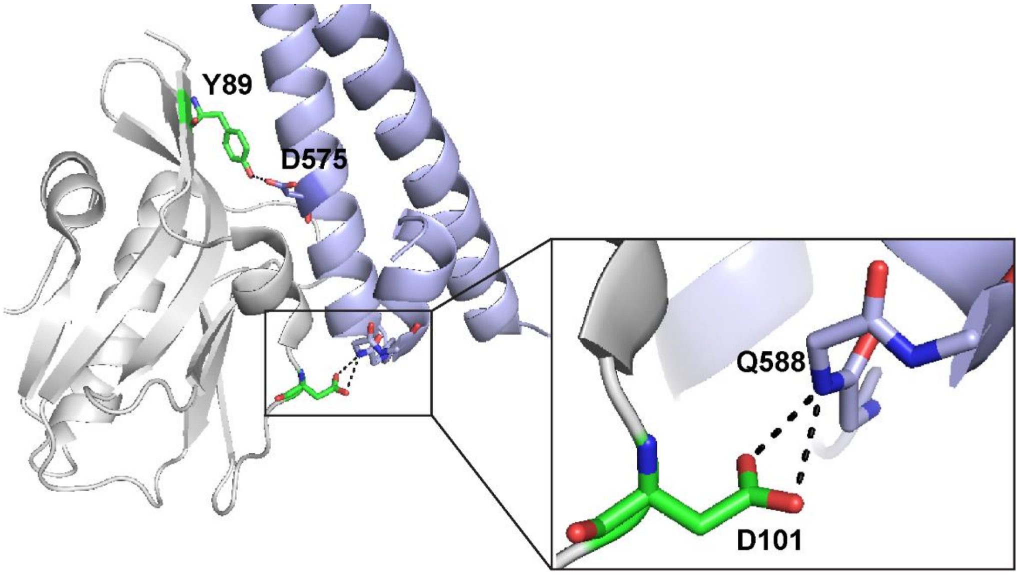

Supplementary Figure 2. Crystal structure of the 1918 NS1:p85β complex (PDB ID: 6U28). 1918 NS1 and p85 are shown in gray and light blue, respectively. Y89 and D101 are shown in green. Inset shows expanded view of D101 interacting with backbone amide of Q588 of p85.

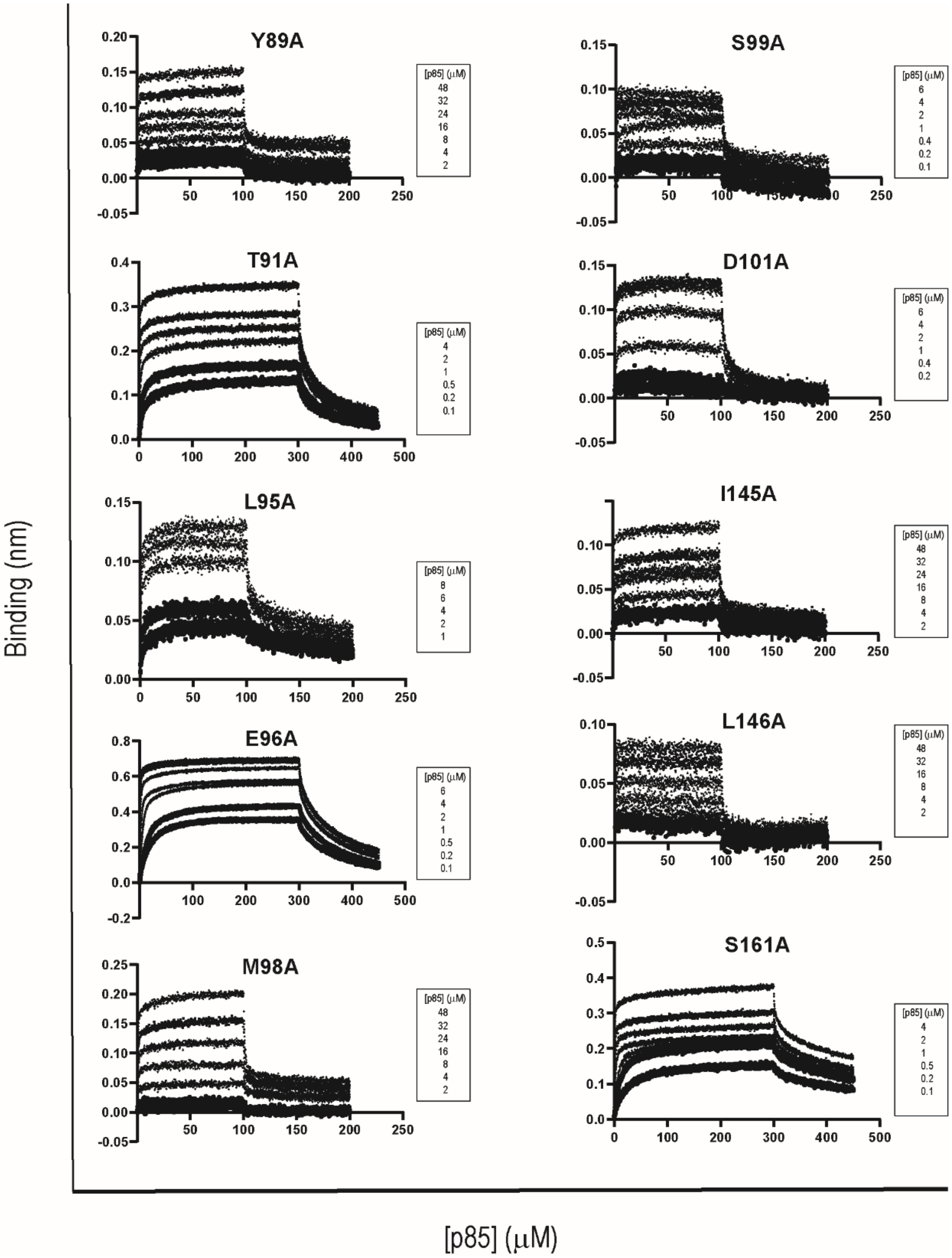

Supplementary Figure 3. Representative BLI sensorgrams of the binding between 1918 NS1 mutants included in the present study and p85β.
